# Effectiveness of different combinations of urea and vermicompost on yield of bitter gourd (*Momordicacharantia*)

**DOI:** 10.1016/j.heliyon.2023.e18663

**Published:** 2023-07-25

**Authors:** Sudip Ghimire, Dhirendra Dhami, Asia Shrestha, Jelisha Budhathoki, Majit Maharjan, Sunil Kandel, Bidhya Poudel Chhetri

**Affiliations:** a Faculty of Agriculture, Agriculture and Forestry University, Rampur, Chitwan, Nepal; b Campus of Live Sciences, Institute of Agriculture and Animal Science, Dang, Nepal

**Keywords:** Flowering, Organic, Production, Transplanting, Vine length

## Abstract

Exclusive use of organic manure in bitter gourd cultivation slows nutrient release, affecting root growth, while inefficient application of fertilizers during transplanting and development stages leads to nutrient losses and increased production costs. The research aimed to evaluate the efficiency of different combinations of urea and vermicompost in bitter gourd production and address the challenges associated with the use of only organic manure. The field experiment conducted in Tulsipur, Dang, utilized a six-treatment randomized complete block design with four replications. The treatments consisted of varying proportions of the recommended dose of urea (16 g plant^−1^) and vermicompost (280 g plant^−1^). Among the treatments, T3 (50% urea and 50% vermicompost) exhibited the most prevalent diameter (3.854 cm), length (16.32 cm), fruit count (1.391), weight plant^−1^ (189.2 g), and weight plot^−1^ (1848 g) while T4 (25% urea and 75% vermicompost) showed the quickest first flowering (30.37 days) and fruiting (33.75 days). T2 (75% urea and 25% vermicompost) recorded the highest vine length (222.5 cm). Statistically significant differences were observed in most recorded characteristics for T3. The results highlight the slow-release nutrient benefits and soil improvement properties of vermicompost, complemented by the quick-release nutrient attributes of urea. The traits with the lowest yield were observed in the control group (T6). A 50% combination of recommended urea dose and vermicompost is recommended for bitter gourd cultivation, leading to improved growth, yield, and quality, underscoring the benefits of organic fertilizer in this situation. The study's findings contribute to the scientific understanding of optimizing fertilization practices in bitter gourd production, minimizing production costs, reducing nutrient losses, and environmental pollution.

## Highlights

• Urea and vermicompost alone reduce nutrient absorption, leading to decreased yield.
• Precise guidelines for fertilizer and manure application are currently lacking.
• The optimal 50% urea and 50% vermicompost combination maximizes bitter gourd yield.
• The recommended combination minimizes production costs and reduces nutrient losses.

## Graphical abstract

**Figure:**
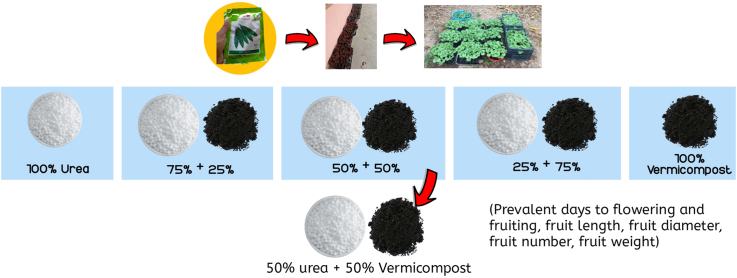

## 1 Introduction

Bitter gourd (*Momordica charantia* L.) is the Neglected and Underutilized Cucurbit Species (NUCuS) of the cucurbitaceae family with somatic chromosome number 22 and is raised for its unripe tuberculate fruits, which have a distinctively bitter flavor [[1], [2], [3], [4], [5], [6], [7]]. It is a lush, climbing vine with somewhat silky stalks adorned with deeply lobed, dark green leaves and yellow dioecious blooms [4,8,9]. Bitter gourd domestication most likely originated in Eastern Asia, particularly in the eastern part of India or the southern zone of China [[10], [11], [12]]. Fruits are used medicinally to treat rheumatism, diabetes, asthma, and blood disorders [[13], [14], [15]]. Extracts of bitter gourd include antioxidant, antimicrobial, antiviral, anti-hepatotoxic, and anti-ulcerogenic properties in addition to being able to lower blood sugar [16,17]. Bitter gourd is also cultivated for ornamental purposes and has wide utilization in traditional medicine [5,18]. Bitter gourd represents a warm-season vegetable that grows well in subtropical as well as hot-arid areas but is vulnerable to mild frost [[19], [20], [21]]. Optimal vine development occurs within a temperature range of 25–30 °C [4] and flourishes in well-drained sandy to sandy loam soils, with a recommended soil pH range of 4.3–8.7 [22]. In the plains, the summer season crop is typically planted between January and February, while the rainy season crop is planted in May. Bitter gourd has a low growth rate against other cucurbitaceae species and requires assistance to flourish.

Management of fertilizer has been recognized as the key element curbing the yield of crops [23]. It is a highly fertilizer-responsive crop and the cultivation of bitter gourd requires an ample supply of plant nutrients [24,25]. Agricultural food production is strongly dependent on the application of chemical fertilizers [26,27]. Nitrogen is essential for plant metabolism, participates in numerous metabolic processes that are very important, and also helps with protein synthesis and serves a vital part in the productivity and quality of crop production [26,28]. The main source of nitrogen utilized in agriculture across the world is urea [29]. When urea is incorporated into the soil, it produces a high quantity of accessible nitrogen early in crop growth that is greater than what the plants need, which might result in N losses and environmental pollution [30].

Organic fertilizers and manures must be used for their proper growth and development. Organic manure like vermicompost improves soil structure as well as increases its water-holding capacity [[31], [32], [33]]. Moreover, it facilitates aeration in the soil. Vermicompost is the end product of a system of decomposition where many types of worms, including red wigglers, white worms, and other earthworms, decompose food scraps, bedding materials, or vegetable waste, and vermicast [34,35]. Given that it contains water-soluble components, it is a fantastic organic fertilizer as well as a soil enhancer that is rich in nutrients [36]. Vermicast is the byproduct of earthworms' decomposing organic matter. It is sometimes referred to as worm castings, worm humus, worm manure, or worm feces. This excreta has been shown to have lower pollution levels and higher nutrient saturation compared to the organic materials utilized for vermicomposting [37]. Soil treated with vermicompost exhibits improved plant development, including more leaves, flowers, and fruits [38]. The addition of vermicompost to the soil enhances its physicochemical characteristics, such as the cations exchange capacity, pH, water, and nutrient preservation, as well as its microbe community [39,40].

Nutrient levels and mineral balances are important management techniques for maximizing crop output and maintaining high market quality [41]. Chemical fertilizers have shown a high response in increasing bitter gourd growth and production, their continuous and heavy application can harm the natural ecology and disrupt nutrient recycling in the soil [42,43]. Furthermore, the cost of chemical fertilizers can be burdensome for small-scale farmers. There is a growing interest in using organic wastes in farming systems to lessen the negative effects of ion alteration [44]. Utilization of organic manure alone in bitter gourd cultivation might cause sluggish nutrient release [45], reducing root, while ineffective fertilizer application during transplanting and development phases results in nutrient losses, environmental hazards, and lower production [26]. Information on the precise guidelines for fertilizer and manure application is currently scarce [46]. Compared to other cucurbits global standards, the yield of bitter is quite low [3], indicating a potential research gap in optimizing production practices. An integrated approach combining organic and inorganic fertilizers is being realized as a sustainable solution to address these challenges. However, studies exploring this approach have been conducted infrequently in South Asia. This study aims to evaluate the optimal combination of urea and vermicompost for maximizing production, ensuring maximum nutrient uptake, and increasing the yield of bitter gourd. The research findings will add both practical and theoretical value to the field of bitter gourd production as the research embraces intra- and inter-fertilizer efficacy analysis and provides valuable recommendations for farmers, enabling them to adopt sustainable and cost-effective practices while increasing yield and reducing nutrient losses.

## 2 Materials and methods

### 2.1 Study site

The experiment took place in April 2022 at the Horticulture Research Farm of Tribhuwan University, Institute of Agriculture and Animal Science, Campus of Live Sciences Tulsipur, Dang, Nepal. The farm was located at 28°07′24.00″N latitude, 82°17′26.40″E longitude, and has an altitude of 647 m above mean sea level (masl). The site lies in the inner Terai region, characterized by a humid subtropical climate. The area receives the majority of its annual rainfall in July and August. The average temperature recorded during the experiment was 20.40 °C.

### 2.2 Soil condition of the field

The experimenting ground's soil was analyzed and determined to be sandy loam with a pH of 6.5, indicating a slightly acidic condition. The soil was characterized by low organic matter content, low nitrogen levels, and medium phosphorous levels.

### 2.3 Cropping history of the field

The experimental field had previously been cultivated with rapeseed and maize during the preceding two seasons. This prior cultivation may have had some influence on the current experiment.

### 2.4 Experimental details

This single factorial study was conducted using the Palee cultivar of bitter gourd. The experiment consisted of six treatments [T1 = Urea (100%) and vermicompost (0%), T2 = Urea (75%) and vermicompost (25%), T3 = Urea (50%) and vermicompost (50%), T4 = Urea (25%) and vermicompost (75%), T5 = Urea (0%) and vermicompost (100%), and T6 = Control], with four replications. The experiment followed a randomized complete block design (RCBD), with a plot size of 16 m^2^ and a total of 24 plots. The row-to-row distance and plant-to-plant distance were both 1 m, while the plot-to-plot distance and distance between replications were both 0.5 m. The date of sowing was February 28th, and transplantation was carried out on March 23rd.

### 2.5 Agronomical operations

#### 2.5.1 Nursery preparation, land preparation, and transplantation

The seedlings were raised in polybags using a soil mixture consisting of coco peat, farmyard manure (FYM), and soil in a ratio of 1.5:1:1. Each polybag included this soil mixture, and a single seed was inserted into each polybag. The primary tillage in the experimental area was carried out using a rotary tiller. For the secondary tillage, manual tools such as spades, hoes, and rakes were utilized. The ground was leveled by breaking up all clods. For transplanting the seedlings, pits measuring 15 × 10 cm^2^ were prepared. After approximately 30 days of sowing the seeds in polybags, the seedlings were transplanted into these pits. In each pit, a base of 12 g each of phosphorus and potassium was placed. Furthermore, the respective doses of urea and vermicompost were added to each pit. The plants were adequately covered with soil after transplantation to ensure that they could access the nutrients available in the soil.

#### 2.5.2 Nutrient management and irrigation

The recommended dose of vermicompost for each plant was 280 g, while the recommended doses of urea, DAP (Di-Ammonium Phosphate), and MOP (Muriate of Potash) were 16:12:12 g plant^−1^. Initially, a basal dose of 6 g of urea was applied, followed by a split dose of 10 g after 25 days of transplantation. However, certain percentages of the recommended doses of urea and vermicompost were applied based on the specific treatment combination. The vermicompost was placed at the bottom of the pit, while the other chemical fertilizers were incorporated using the ring method. Light irrigation was provided shortly following sowing to encourage growth and germination. Subsequently, additional irrigations were given at intervals of 4 days.

#### 2.5.3 Weeding, plant protection measures, and harvesting

Hand weeding was performed twice during the experiment, first after 25 days and then 5 days after transplantation. A supplement consisting of gibberellic acid (GA_3_) and a mixture of calcium and boron was added to accelerate plant proliferation and development. Cue lures were strategically placed in different locations within the field to protect the fruits from fruit flies. The experimental data were collected separately, and subsequently, the fruits were harvested to record the yield plot^−1^. Four harvesting sessions took place on the following dates: May 29th, June 5th, June 12th, and June 19th.

### 2.6 Data collection of parameters

Out of 16 plants, the middle 8 plants were used for data collection.

#### 2.6.1 Days to first flowering and fruiting, and vine length

Regular visits to the experimental field were conducted to observe the first flowering and fruiting of each plant. The vine length was measured twice using a measuring tape, specifically at 45 and 60 days after transplantation (DAT).

#### 2.6.2 Fruit count plant^−1^, fruit length, fruit diameter, average fruit weight plant^−1^, and average fruit weight plot^−1^

The first harvest took place 48 DAT, where the middle 8 plants of each plot were selected. The fruits count plant^−1^ were counted individually. Subsequent harvests were conducted at 7-day intervals. For each harvest, the fruit lengths of the middle 8 plants were measured using a scale, and the diameter of every single fruit was determined using vernier calipers. The average harvested fruit weight from the middle 8 plants in each plot was recorded using a weighing machine, and the fruit weight plant^−1^ was calculated. The mean fruit weight obtained from each plot was calculated by weighing the total yield of the plot.

### 2.7 Statistical analysis

The obtained raw data from various parameter readings were first tabulated in MS Excel 2010 and were analyzed statistically using GenStat 15th edition. Duncan's Multiple Range Test (DMRT) was used to ascertain the significant differences between the mean values at a 5% level of significance.

## 3 Results and discussion

### 3.1 Days to first flowering and fruiting

The first day of flowering showed significant variations due to different treatments (Table 1). Among the treatments, T4 (urea 25% and vermicompost 75%) had the earliest first flower at 30.37 days, followed by T2 (urea 75% and vermicompost 25%) at 30.94 days, T5 (urea 0% and vermicompost 100%) at 32.44 days, T3 (urea 50% and vermicompost 50%) at 32.69 days, and T1 (urea 100% and vermicompost 0%) at 35.19 days. The longest time to flowering was observed in T6 (Control) at 38.31 days.

**Table 1:** Urea and vermicompost effects on the bitter gourd's first flowering and fruiting.

Treatments	Details (% of recommended dose)	Days to first flowering	Days to first fruiting
T1	Urea (100%) and vermicompost (0%)	35.19^b^	38.69^c^
T2	Urea (75%) and vermicompost (25%)	30.94^a^	34.88^ab^
T3	Urea (50%) and vermicompost (50%)	32.69^ab^	36.25^b^
T4	Urea (25%) and vermicompost (75%)	30.37^a^	33.75^a^
T5	Urea (0%) and vermicompost (100%)	32.44^ab^	35.81^ab^
T6	Control	38.31^c^	44.25^d^
Grand Mean		33.32	37.27
SEm (±)		1.273	1.016
Probability		0.001**	0.001**
L.S.D.		2.714	2.164
CV%		5.4	3.9

Columns with the same letters in DMRT are not significantly different (p = 0.05), SEm(±) = Standard Errors of Means, CV = Coefficient of Variation, LSD = Least Significant Difference, ** = highly significant difference at p < 0.01.

Significant variations in the first day of fruiting were exhibited over the different treatments, indicating that the treatments had a significant effect on the time it took for fruits to appear (Table 1). T4 (urea 25% and vermicompost 75%) exhibited the shortest duration to first fruiting at 33.75 days, followed closely by T2 (urea 75% and vermicompost 25%) at 34.88 days. T5 (urea 0% and vermicompost 100%) took 35.81 days to reach first fruiting, while T3 (urea 50% and vermicompost 50%) showed a slightly longer duration of 36.25 days. T1 (urea 100% and vermicompost 0%) experienced a further delay, with the first fruiting occurring after 38.69 days. Notably, the longest time to first fruiting was observed in T6 (Control), which required 44.35 days.

When compared to applying the nutrients singly, applying different mixtures of organic and inorganic nutrient sources to plants had a bigger influence on their height. During the initial stages of growth, when the plant possesses an underdeveloped root system and a limited number of branches and leaves, the application of urea alone can result in a higher loss of nutrients and inadequate utilization by the plant [26,47]. However, when vermicompost is added, it releases nutrients gradually, providing the plant with sufficient time to absorb and utilize the nutrients effectively [48]. Vermicompost enriches the soil by boosting microbial activity, and improving aeration, infiltration, and water retention [49,50]. These enhancements facilitate enhanced root development and overall vegetative growth [51,52]. The absorption of nutrients, notably nitrogen, which promoted cell division and cell elongation and resulting in rapid growth, and since nitrogen is freely available to plants in an equal mix of fertilizer sources. Consequently, T4 exhibited the earliest flowering and fruiting stages. In contrast, the absence of nutrient sources in treatment T6 resulted in delayed flowering and fruiting stages. Conversely, in T1, where the nutrient loss occurred and soil quality for root development was inferior to the treatment involving vermicompost application, the flowering process may experience delays, consequently leading to an extended duration for fruiting. Shree et al. [53] discovered that using 100% vermicompost led to a significant reduction in the time taken for the first flowering. Similar positive effects were observed with vermicompost at 75% and 50% levels. Utilizing vermicompost not only improved soil physical properties but also fostered the development of a robust root system, resulting in enhanced nutrient and water absorption. These factors ultimately contributed to accelerated plant growth and earlier onset of flowering and fruiting [54]. The findings resonated with the results reported by Sreenivas et al. [55], and Kameswari et al. [56] in their studies on ridge gourd as well as by Prashanthi et al. [57] on bitter gourd.

### 3.2 Fruit count plant-1

The analysis of the data indicated a significant finding regarding the fruits count, specifically concerning the different treatments applied (Table 2). This suggests that the treatments have a substantial effect on the duration required for fruit production. Among the different treatments, T3 (urea 50% and vermicompost 50%) showcased the highest fruits count plant-1 (1.391) closely pursued by T2 (urea 75% and vermicompost 25%) with a record value of 1.281. T4 (Urea 25% and vermicompost 75%) yielded a slightly lower fruit count (1.219), while T5 (urea 0% and vermicompost 100%) displayed a diminished fruit count (0.875). T1 (Urea 100% and vermicompost 0%) resulted in a moderate fruits count (0.805), whereas the control group (T6) exhibited the least fruit production at 0.097. Similar results were found by Prashanthi et al. [57] that application of 50% NPK + 50% Vermicompost significantly increases fruits count plant^−1^.

**Table 2:** Urea and vermicompost effects on the bitter gourd's fruits plant^−1^.

Treatments	Details (% of recommended dose)	Fruits count plant^−1^
T1	Urea (100%) and vermicompost (0%)	0.805^b^
T2	Urea (75%) and vermicompost (25%)	1.281^ab^
T3	Urea (50%) and vermicompost (50%)	1.391^a^
T4	Urea (25%) and vermicompost (75%)	1.219^ab^
T5	Urea (0%) and vermicompost (100%)	0.875^b^
T6	Control	0.097^c^
Grand Mean		0.945
SEm (±)		0.2156
Probability		0.001**
L.S.D.		0.4595
CV%		32.3

Columns with the same letters in DMRT are not significantly different (p = 0.05), SEm(±) = Standard Errors of Means, CV = Coefficient of Variation, LSD = Least Significant Difference, ** = highly significant difference at p < 0.01.

The observed higher fruit count in T3 could be attributed to better root development and improved nutrient uptake during the early stages, as well as increased nitrogen availability during the reproductive phase. The application of a split dose of fertilizers might have played a role in enhancing fruit production in this treatment [58]. Better root development and efficient nutrient uptake in the earlier stage and higher nitrogen availability during the reproductive phase or fruiting in the form of split dose might have resulted in higher fruit count in T3. Vermicompost makes sufficient amounts of plant nutrients available throughout the growth period and especially at critical growth periods of crops resulting in better uptake, plant vigor, as well as enhanced yield qualities [59]. The application of nitrogen, phosphorus, and potassium through organic manures, for example, may have sped up the production of chlorophyll and amino acids and increased the translocation of photosynthesis from leaves to fruits, which increased the fruit count plant^−1^. Urea provides the rapidly available nitrogen essential for the precise advancement and development of the vine to produce fruit [60].

### 3.3 Average vine length

The results indicate that the treatments had a significant impact on the average vine length. The highest vine length was observed in T2 (urea 75% and vermicompost 25%), measuring 225.5 cm. This was followed by T4 (urea 25% and vermicompost 75%) at 222.9 cm and T3 (urea 50% and vermicompost 50%) at 218.5 cm. T5 (urea 0% and vermicompost 100%) showed a slightly lower vine length of 205.9 cm. T1 (urea 100% and vermicompost 0%) exhibited a vine length of 202.1 cm. The minimum vine length of 116.4 cm was observed in T6 (control), as illustrated in Table 3.

**Table 3:** Urea and vermicompost effects on the bitter gourd's vine length.

Treatment	Detail (percentage of recommended dose)	Vine length
T1	Urea (100%) and vermicompost (0%)	202.1^a^
T2	Urea (75%) and vermicompost (25%)	225.5^a^
T3	Urea (50%) and vermicompost (50%)	218.5^a^
T4	Urea (25%) and vermicompost (75%)	222.9^a^
T5	Urea (0%) and vermicompost (100%)	205.9^a^
T6	Control	116.4^b^
Grand Mean		198.6
SEm (±)		23.05
Probability		0.002**
L.S.D		49.13
CV%		16.4

Columns with the same letters in DMRT are not significantly different (p = 0.05), SEm(±) = Standard Errors of Means, CV = Coefficient of Variation, LSD = Least Significant Difference, ** = highly significant difference at p < 0.01.

This could be due to an increase in nutrient supply caused by a specific combination of integrated nutrient management treatments, which delivered enough nitrogen, which is linked to strong photosynthetic activity, root development, and increased carbohydrate translocation from source to growing points. Profound vine growth was seen at the time when a split dose of urea is given [58]. A higher split dose of urea combined with better root development due to vermicompost at an earlier stage of the plant might have led to the highest vine length in T2. The utilization of vermicompost treatments, with their higher organic carbon, nitrogen, and phosphorus content, resulted in significantly greater vine length compared to sole urea usage [61].

### 3.4 Average fruit length and diameter (cm)

The treatment that exhibited the maximum fruit length was T3 (urea 50% and vermicompost 50%), reaching 16.32 cm, followed by T5 (urea 0% and vermicompost 100%) at 16.16 cm, T4 (urea 25% and vermicompost 75%) 16.01 cm as shown in Table 3. Notably, T2 (urea 75% and vermicompost 25%) exhibited a fruit length of 15.57 cm, while T1 (urea 100% and vermicompost 0%) demonstrated a slightly lower length of 13.99 cm. The control treatment, T6, recorded the minimum fruit length among all treatments, measuring 10.64 cm.

The data analysis for average fruit diameter revealed non-significance, indicating that the treatments applied did not have a significant effect on fruit diameter (Table 4). The treatment T3 (urea 50% and vermicompost 50%) showcased a maximum fruit diameter of 3.854 cm. Following closely, T5 (urea 0% and vermicompost 100%) achieved a diameter of 3.803 cm, while T4 (urea 25% and vermicompost 75%) attained a noteworthy diameter of 3.776 cm. Notably, T1 (urea 100% and vermicompost 0%) exhibited a slightly smaller fruit diameter of 3.712 cm, and T2 (urea 75% and vermicompost 25%) demonstrated a diameter of 3.636 cm. Among all treatments, the control group (T6) recorded the minimum diameter, measuring 3.461 cm.

**Table 4:** Urea and vermicompost effects on the bitter gourd's fruit length and diameter.

Treatments	Details (% of recommended dose)	Average fruit length	Average fruit diameter
T1	Urea (100%) and vermicompost (0%)	13.99^b^	3.712^ab^
T2	Urea (75%) and vermicompost (25%)	15.57^a^	3.636^ab^
T3	Urea (50%) and vermicompost (50%)	16.32^a^	3.854^a^
T4	Urea (25%) and vermicompost (75%)	16.01^a^	3.776^ab^
T5	Urea (0%) and vermicompost (100%)	16.16^a^	3.803^a^
T6	Control	10.64^c^	3.461^b^
Grand Mean		14.78	3.707
SEm (±)		0.699	0.1399
Probability		<0.001***	NS
L.S.D.		1.490	0.2981
CV%		6.7	5.3

Columns with the same letters in DMRT are not significantly different (p = 0.05), SEm(±) = Standard Errors of Means, CV = Coefficient of Variation, LSD = Least Significant Difference, *** = very significantly different at p < 0.001, NS = Non-significant.

The treatment, T3 exhibited superior fruit length and diameter, possibly due to the initial nitrogen dose from urea and vermicompost, which stimulated deep root growth. This led to increased nutrient absorption, promoting fruit growth and development. The use of mixed fertilizers enables bitter gourd crops to have improved nutrient accessibility compared to individual fertilizers [62]. Conversely, T6 displayed the smallest fruit length and diameter, likely due to insufficient nitrogen availability required for optimal fruit growth. Similar results were found by Prashanthi et al. [57] that application of 50% NPK + 50% vermicompost significantly increases the fruit measurements. The enhanced fruit length and diameter observed in T3 could be attributed to the presence of abundant nutrients in the organic fertilizers, which are released slowly and effectively taken up by the crops. Utilizing vermicompost helped mitigate nutrient loss and leaching, thereby improving nutrient utilization and ultimately contributing to increased fruit measurements [63]. This gradual increase in fruit measurements may have been caused by the combined use of vermicompost and urea.

### 3.5 Average fruit weight plant-1, and fruit weight plot-1 (g)

The analysis of the average fruit weight plant^−1^ and fruit weight plot^−1^ indicates a significant influence of the treatments (Table 5). The highest maximum fruit weight plant^−1^ and plot^−1^ were observed in treatment T3 (urea 50% and vermicompost 50%), measuring 189.2 g and 1848 g, respectively, followed by treatment T4 (urea 25% and vermicompost 75%) with values of 168.5 g and 1660 g. Treatment T3 (urea 75% and vermicompost 25%) yielded a fruit weight plant^−1^ of 163.6 g and a fruit weight plot^−1^ of 1560 g. Treatment T1 (urea 100% and vermicompost 0%) resulted in the fourth-highest fruit weight plant^−1^, which was 127.9 g, followed by T5 (urea 0% and vermicompost 100%) with a value of 126.4 g. Treatment T5 (urea 0% and vermicompost 100%) also resulted in the fourth-highest fruit weight plot^−1^, amounting to 1084 g, followed by T1 (urea 100% and vermicompost 0%) with a value of 864 g. The lowest fruit weight plant^−1^ and fruit weight plot^−1^ were observed in T6 (control), measuring 23.1 g and 92 g, respectively.

**Table 5:** Urea and vermicompost effects on the bitter gourd's fruit weight plant^−1^ and fruit weight plot^−1^.

Treatments	Details (% of recommended dose)	Average fruit weight plant^−1^	Average fruit weight plot^−1^
T1	Urea (100%) and vermicompost (0%)	127.9^b^	864^b^
T2	Urea (75%) and vermicompost (25%)	163.6^ab^	1570^a^
T3	Urea (50%) and vermicompost (50%)	189.2^a^	1848^a^
T4	Urea (25%) and vermicompost (75%)	168.5^a^	1660^a^
T5	Urea (0%) and vermicompost (100%)	126.4^b^	1848^b^
T6	Control	23.1^c^	92^c^
Grand Mean		133.1	1186
SEm (±)		17.21	187.9
Probability		0.001**	0.001**
L.S.D.		36.67	400.4
CV%		18.3	22.4

Columns with the same letters in DMRT are not significantly different (p = 0.05), SEm(±) = Standard Errors of Means, CV = Coefficient of Variation, LSD = Least Significant Difference, ** = highly significant difference at p < 0.01.

Similar results were found by Prashanthi et al. [57] that application of 50% NPK + 50% vermicompost significantly increases the weight of fruits, as well as by Ebrahimi et al. [52] where 50% vermicompost mixed with biochar resulted in the highest yield in eggplants compared to the amendment of sole fertilizers in any proportion [52]. The observed variations in fruit weight among the treatments may be attributed to improved root development and efficient nutrient uptake during the early growth stages, as well as higher nitrogen availability during the reproductive phase or fruiting, achieved through split dose application [58]. The utilization of vermicompost, which provides a steady supply of plant nutrients throughout the growth period and critical stages, coupled with urea, contributes to increased fruit yield, improved nutrient uptake, and enhanced plant vigor [[31], [32], [33]] as well as boosted the magnesium, nitrogen, and zinc uptake by raising the pH of the soil [40,52,64], so the combination of urea and vermicompost results in higher fruit production. T4 resulted in a higher yield in both the plants and plots, as its yield attributing characteristics directly contributed to the increased fruit yield.

However, various factors, including cultivar, agronomic factors (such as irrigation, planting methods, and spacing), and climatic factors (such as temperature, relative humidity, growing season, rainfall, and soil fertility), could have influenced the outcomes of the production process, potentially leading to different results [20,[65], [66], [67], [68], [69]]. Since the study was conducted in specific locations, the findings may not directly apply to other cultivation areas due to variations in soil characteristics and other factors.

## 4 Conclusion and recommendations

The combination of a 50% recommended dose of urea (8 g plant^−1^) and 50% vermicompost (140 g plant^−1^) yields favorable results for bitter gourd cultivation. Among the treatments, T3 exhibited the highest diameter (3.854 cm), length (16.32 cm), fruit count (1.391), weight plant^−1^ (189.2 g), and weight plot^−1^ (1848 g). Statistically significant differences were observed in most recorded characteristics for T3, highlighting the effectiveness of this treatment. The yield differences were minimal among the various urea and vermicompost combinations employed, indicating that any combination is superior to sole usage. The utilization of vermicompost, providing a steady supply of plant nutrients throughout the growth period and critical stages, in conjunction with urea, contributes to increased fruit yield, improved nutrient uptake, and enhanced plant vigor. The results emphasize the slow-release nutrient benefits and soil improvement properties of vermicompost, complemented by the quick-release nutrient attributes of urea. This combination enhances growth, yield, and quality attributes, underscoring the advantages of incorporating organic manure. Furthermore, the study emphasizes the importance of optimizing fertilization practices in bitter gourd production to minimize costs, reduce nutrient losses, and mitigate environmental pollution. However, the outcomes of the production process could have been influenced by various factors, such as different cultivars, single season cultivation, agronomic practices, climatic conditions, and the use of open fields for research, which may be susceptible to the presence of biotic organisms. These variables have the potential to impact the results significantly, potentially yielding diverse outcomes. Future research should consider multifactorial studies examining the effects of different cultivars, agronomic factors as well as climatic factors on the production process to gain a more comprehensive understanding. Researchers can acquire more precise insights into their individual and combined impacts, leading to a more comprehensive understanding of the subject matter.

## Author contribution statement

Sudip Ghimire, Dhirendra Dhami, Asia Shrestha, Majit Maharjan, Sunil Kandel, Jelisha Budhathoki, Bidhya Poudel Chhetri: Conceived and design the experiments; Performed the experiments; Analyzed and interpreted the data; Contributed reagents, materials, analysis tools or data; Wrote the paper.

## Data availability statement

Data will be made available on request.

## Funding details

This research did not receive any specific grant from funding agencies in the public, commercial, or not-for-profit sectors.

## Declaration of competing interest

The authors declare that they have no known competing financial interests or personal relationships that could have appeared to influence the work reported in this paper.
